# Endoluminal Vacuum Therapy (E-Vac): A Treatment Option in Oesophagogastric Surgery

**DOI:** 10.1007/s00268-018-4463-7

**Published:** 2018-01-25

**Authors:** D. J. Pournaras, R. H. Hardwick, P. M. Safranek, V. Sujendran, J. Bennett, G. D. Macaulay, A. Hindmarsh

**Affiliations:** Cambridge Oesophago-Gastric Centre, Addenbrooke’s Hospital, Cambridge University Hospitals, Hills Rd, Cambridge, CB2 0QQ UK

## Abstract

**Background:**

Leaks from the upper gastrointestinal tract often pose a management challenge, particularly when surgical treatment has failed or is impossible. Vacuum therapy has revolutionised the treatment of wounds, and its role in enabling and accelerating healing is now explored in oesophagogastric surgery.

**Methods:**

A piece of open cell foam is sutured around the distal end of a nasogastric tube using a silk suture. Under general anaesthetic, the foam covered tip is placed endoscopically through the perforation and into any extra-luminal cavity. Continuous negative pressure (125 mmHg) is then applied. Re-evaluation with change of the negative pressure system is performed every 48–72 h depending on the clinical condition. Patients are fed enterally and treated with broad-spectrum antibiotics and anti-fungal medication until healing, assessed endoscopically and/or radiologically, is complete.

**Results:**

Since April 2011, twenty one patients have been treated. The cause of the leak was postoperative/iatrogenic complications (14 patients) and ischaemic/spontaneous perforation (seven patients). Twenty patients (95%) completed treatment successfully with healing of the defect and/or resolution of the cavity and were subsequently discharged from our care. One patient died from sepsis related to an oesophageal leak after withdrawing consent for further intervention following a single endoluminal vacuum (E-Vac) treatment. In addition, two patients who were successfully treated with E-Vac for their leak subsequently died within 90 days of E-Vac treatment from complications that were not associated with the E-Vac procedure. In two patients, E-Vac treatment was complicated by bleeding. The median number of E-Vac changes was 7 (range 3–12), and the median length of hospital stay was 35 days (range 23–152).

**Conclusions:**

E-Vac therapy is a safe and effective treatment for upper gastrointestinal leaks and should be considered alongside more established therapies. Further research is now needed to understand the mechanism of action and to improve the ease with which E-Vac therapy can be delivered.

## Introduction

The treatment of upper gastrointestinal tract perforations and postoperative anastomotic leaks remains challenging. The anastomotic leak rate after oesophagectomy and gastrectomy in England and Wales is 5 and 7%, respectively [1]. Laparoscopic surgery for obesity is associated with a leak rate of 0.6% after primary gastric bypass, 0.8% after primary sleeve gastrectomy, and 1.3% for revisional surgery [2]. Iatrogenic perforations after diagnostic endoscopy are rare (0.05%), but more common after therapeutic interventions (2.6%) [3, 4]. The principles of managing a perforation or anastomotic leak depend on the cause, the size of the defect, the time from the leak to treatment, and the physiological status of the patient. Management includes providing physiological support (usually in a level II or III facility), adequate drainage of any extra-luminal collections, systemic antibiotics and anti-fungal therapy, and maintenance of enteral nutrition. It may be appropriate to consider occlusion of a gastrointestinal defect using a stent but these often migrate and can erode adjacent structures such as the airway when placed in the oesophagus [5, 6]. Creation of an external fistula using a T-tube, or excision of the ischaemic tissue and diversion of luminal contents are well established strategies but surgical intervention is a high risk strategy when there has been delay in diagnosis and patients are profoundly septic, especially if the intervention requires one-lung ventilation.

Negative-pressure wound therapy has revolutionised the treatment of complex infected wounds [7]. There have been reports in the literature of using the same principle within the GI tract after surgery [8–22]. Endoluminal vacuum therapy has been used successfully to treat anastomotic leaks after colorectal surgery [23, 24] and oesophagectomy [8, 9]. We report our experience of introducing endoluminal vacuum (E-Vac) therapy in a regional oesophagogastric centre in the UK.

## Materials and methods

The procedure is performed in the operating theatre or intensive treatment unit with the patient anaesthetised and intubated. General anaesthesia is required to facilitate insertion of the E-Vac device, which is performed using a standard diagnostic gastroscope. After initial endoscopic assessment of the defect, a nasogastric tube is passed through the nose and out through the patients mouth. A 4 × 0.5 cm piece of open cell foam is cut from a standard sponge (V.A.C.--^®^ GranuFoam™ Small Dressing Kit, KCI) so that it is just large enough to cover all the holes at the end of the nasogastric tube. The foam is wrapped around the end of the nasogastric tube and sutured in place with four interrupted 0 silk transfixion sutures, the most distal of which is left slightly long to create a loop. This is grasped with endoscopic biopsy forceps through the working channel of the gastroscope. The silk loop is withdrawn into the working channel so that the E-Vac device sits side by side with the gastroscope and the two together are then manipulated under vision into the pharynx and through cricopharyngeus. The scope is advanced pulling the E-Vac device with it until the defect is reached. The biopsy forceps are then used to advance the sponge beyond the scope and into the defect. As soon as this is achieved, the external end of the nasogastric tube is connected to a suction pump and a negative pressure of 125 mm Hg applied to anchor the device within the cavity. The scope is then removed. The procedure is repeated every 48–72 h depending on the clinical response. If the patient does not already have a surgically placed feeding jejunostomy, one can be inserted at that time.

In all patients, E-Vac therapy was continued until sepsis was controlled, and the leak cavity was lined with healthy granulation tissue with no significant dependent component. The healing response of the leak cavity to treatment was assessed endoscopically each time the E-Vac device was removed.

## Data collection

Data were collected prospectively including source of referral, patient demographics, indication for endoscopic treatment, the number of E-Vac changes required to successfully treat the leak cavity, hospital stay, complications and mortality.

## Results

Since April 2011, twenty one patients have been treated with E-Vac therapy at our Centre. Five patients were tertiary referrals from other institutions. Of the 21 patients, seven had an anastomotic leak, seven had an iatrogenic perforation following an endoscopic or non-resectional surgical procedure and seven had a spontaneous or ischaemic perforation. All patients we treated with iatrogenic perforations either were delayed presentations, some having failed alternative treatment strategies, or were too unwell to undergo immediate surgical treatment. A detailed description of the indication and anatomical site of the leak cavity for individual patients is shown in Table 1.

**Table 1:** Indications for E-Vac

Indication for treatment
Anastomotic leak after thoracoscopically assisted three-stage oesophagectomy
TOE-related oesophageal perforation (upper third) during emergency mitral valve repair
Perforated gastric antral ulcer
Leak at gastro-gastric anastomosis after multivisceral transplant including the small bowel
Gastric perforation secondary to ischaemic necrosis on a background of giant hiatus hernia
Posterior gastric wall perforation after subtotal pancreatectomy and splenectomy for IPMN
Spontaneous rupture of the oesophagus
Leak at gastro-gastric anastomosis after multivisceral transplant including the small bowel
Duodenal necrosis
Gastric perforation secondary to pancreatic stent insertion
Iatrogenic tear of the GOJ during mobilisation, open revisional hiatal surgery
Anastomotic leak after emergency total gastrectomy for acute herniation of giant hiatal hernia
Oesophageal perforation (middle third) during lung transplant for cystic fibrosis
Fistula between gastric conduit and lung, previous oesophagectomy
Gastric necrosis secondary to pancreatitis
Iatrogenic oesophageal perforation (upper third) during ENT rigid oesophagoscopy
Anastomotic leak after Ivor-Lewis oesophagectomy
Iatrogenic oesophageal (upper third) perforation during OGD
TOE-related oesophageal perforation (upper third) during emergency AVR
Anastomotic leak after extended total gastrectomy
Anastomotic leak after left thoracoabdominal oesophagectomy

The median number of E-Vac changes was 7 (range 3–12), and the median length of hospital stay was 35 days (range 23–152). The length of stay reflects the long hospitalisation of these patients due to their initial underlying pathology and not the length of the E-Vac treatment exclusively.

Twenty patients (95%) completed treatment successfully with healing of the defect and/or resolution of the cavity and were subsequently discharged from our care. One 82-year-old patient died after withdrawing consent for further intervention following one E-vac treatment. She was transferred to our Centre following an emergency mitral valve repair in another institution during which time her oesophagus was perforated by a trans-oesophageal echocardiogram probe. The diagnosis was made 10 days after the injury occurred. Her initial response to E-Vac therapy was encouraging, but following withdrawal of her consent to further treatment, she was transferred to a hospice where she died. A further two patients died within 90 days of starting of E-Vac therapy. Both patients had successfully completed E-Vac therapy with resolution of their leak but died of non-leak related causes. One, a 51-year-old patient, died after an anastomotic leak at the gastro-gastric anastomosis following a small bowel transplant. Sepsis was controlled with E-Vac therapy and the defect closed completely, but the patient died from bone marrow failure secondary to his immunosuppression treatment 2 months later. The second patient, aged 48 years, underwent a bilateral lung transplant for cystic fibrosis but developed a mediastinal abscess. This was drained surgically at the primary institution, which resulted in an oesophageal perforation. This was repaired with an intercostal muscle flap which subsequently failed. The patient was transferred to our Centre, and E-Vac therapy commenced. The perforation closed after 10 weeks. The patient started eating again and was transferred back to the referring hospital, but developed a fatal pulmonary embolus within 90 days.

There were two complications (10%) associated with E-Vac treatment, both due to bleeding. The first patient had a posterior gastric perforation secondary to pancreatitis. This was treated with E-Vac therapy, and when the patient bled, a laparotomy was performed to control the bleeding and repair the perforation. The patient went on to have additional E-Vac therapy following a further leak from the surgical repair and made a full recovery. The second patient was treated for an anastomotic leak following an Ivor-Lewis oesophagectomy. A direct aortic branch communicated with the cavity causing significant bleeding. This was controlled with a covered aortic stent. The patient made a full recovery and was discharged home after 54 days.

## Discussion

A number of case reports and series of patients treated with E-Vac therapy have been published to date, all supporting the hypothesis that it is a safe and effective technique for managing upper gastrointestinal leaks [8–22]. Here, we present our initial experience with E-Vac therapy. Our practice has evolved over time. Initially, we considered E-Vac therapy only when all other conventional treatment options had failed or were deemed impossible. However, having gained confidence in its safety and efficacy, we are now using it a first-line treatment in our management algorithms for all upper gastrointestinal perforations and anastomotic leaks in preference to other interventions such as endoscopic stenting or surgery.

One of the major concerns regarding the use of E-Vac therapy is the risk of bleeding. Leak cavities in the upper gastrointestinal tract are often situated in the vicinity of major vessels in the either mediastinum, or lesser sac/retroperitoneum of the upper abdomen. However, we did not have a significant issue with bleeding in this case series. Bleeding did occur in two patients during E-Vac treatment, one from the pancreas during treatment of a posterior gastric perforation caused by acute severe pancreatitis, and the second from a small aortic branch during treatment of an anastomotic leak after an Ivor-Lewis oesophagectomy. In both cases, it was obvious the patient was bleeding as fresh blood was evident in the E-Vac output fluid. E-Vac treatment was terminated immediately. The patients subsequently underwent surgery and aortic stenting, respectively, to prevent further bleeding. Based on our experience, the proximity of a leak cavity to a major vessel is not a contraindication to E-Vac treatment. However, we would recommend a triple-phase CT scan of the anatomical area of interest prior to starting treatment to exclude vascular issues, which may contribute to bleeding such as the development of a pseudoaneurysm. If a significant bleed does occur during treatment, the vacuum should be removed from the E-Vac device, and a triple-phase CT performed to direct subsequent management.

Routine changing of the E-Vac device is not usually associated with bleeding in the leak cavity. Occasionally, due to granulation tissue ingrowth into the E-Vac sponge, minor bleeding can occur during removal of the sponge. In our experience, this is self-limiting and we would reapply the E-Vac directly. More frequent changes of the E-Vac device reduce the risk of this happening.

The patients in our case series have a number of disparate causes for their upper gastrointestinal leaks, which reflects the specialised services provided in our Centre. We have not used this technique for complications associated with bariatric surgery as this type of surgery is not routinely performed in our Centre. However, E-Vac treatment has been described for the management of staple line leaks after sleeve gastrectomy and is likely to be of value in managing this challenging problem, highlighting the importance of the transfer of skills and techniques between the two branches of oesophaghogastric surgery [17, 25]. The heterogeneity of our patients suggests that the technique is useful irrespective of the cause of the mucosal defect.

The mechanism of action is likely to be multifactorial. The negative pressure applied to the leak cavity facilitates effective drainage of extra-luminal sepsis as well as achieves source control by collapsing the cavity around the sponge and occluding the site of the leak. Continuous apposition of the cavity walls results in obliteration of the cavity as they adhere to each other. Extrapolating from the mechanisms of action of wound vacuum therapy, the negative pressure generated by E-Vac therapy may also accelerate tissue healing and repair by modulation of the cytokine response and angiogenesis mechanisms [26]. We observed a good outcome in patients even when the sponge could not be placed (for one or two changes) in the cavity itself and was placed in the gastrointestinal lumen adjacent to the mucosal defect. This suggests that direct contact between the extra-luminal cavity and the sponge is not critical.

Our observation is that once the extra-luminal cavity related to the perforation/leak is lined by healthy granulation tissue, sepsis controlled and there is no large dependent component to the cavity, E-Vac therapy can be discontinued. We did not use alternative or adjunct endoscopic methods to manage the leaks in this series. Our criteria for stopping E-Vac therapy were clinical stability with sepsis control and good drainage of the cavity into the lumen. Patients were then allowed to take free oral fluids and their condition monitored carefully for 3–5 days before allowing soft diet if they remained well. Enteral tube feeding was continued until full oral intake resumed, and antibiotics and anti-fungal agents stopped when the C-reactive protein levels are 10 or less.

Nutrition remains a crucial pillar of E-Vac treatment. We aimed to establish enteral feeding as soon as it was safe and feasible to do so. Although enteral nutrition can be achieved using a nasojejunal feeding tube or percutaneous endoscopic gastrostomy (PEG), surgical access with feeding jejunostomy is our preferred option, depending on the site of the perforation. This method of feeding was utilised in all the patients in our series with no related complications. Parenteral nutrition should be established in the first instance as a bridge until enteral feeding can be established, and can be used as the mainstay of nutritional support if enteral feeding is contraindicated.

As this is a novel technique, using the IDEAL framework, this is phase 2b (exploration) [27]. This occurs when a new procedure has been described and the main technical aspects worked out. Larger numbers of patients are usually needed (up to a few hundred) before a randomised clinical trial that compares the new procedure with traditional management is feasible. Although E-Vac therapy can be used in cases with no other alternatives, if this technique was to be become first-line treatment for the management of upper gastrointestinal leaks, then the next stage (stage 3) would be to design a randomised control trial. There are already four comparisons between E-Vac therapy and self-expanding metal stents favouring E-Vac therapy in terms of both effectiveness and lower adverse event rate [5, 6, 28, 29]. In the meantime, and due to the fact that the population treated is very heterogeneous, a registry at national and international level would be very useful to collect efficacy and safety data. From a technology evolution standpoint, the next step would be the development of a “through the scope” system allowing the use of this device without the need of tracheal intubation.

Our experience to date leads us to conclude that E-Vac therapy is a safe and effective treatment for managing upper gastrointestinal perforations and leaks. The development of a “through the scope” device with deployment of the sponge directly into the leak cavity would greatly simplify its application, negate the need for general anaesthesia and allow standardisation of the technique which would facilitate clinical trials.
